# Use of Sieving as a Valuable Technology to Produce Enriched Buckwheat Flours: A Preliminary Study

**DOI:** 10.3390/antiox8120583

**Published:** 2019-11-25

**Authors:** Beatriz Martín-García, Federica Pasini, Vito Verardo, Ana María Gómez-Caravaca, Emanuele Marconi, Maria Fiorenza Caboni

**Affiliations:** 1 Department of Analytical Chemistry, Faculty of Sciences, University of Granada, Avd. Fuentenueva s/n, 18071 Granada, Spain; bearu15@correo.ugr.es (B.M.-G.); anagomez@ugr.es (A.M.G.-C.); 2 Department of Agricultural and Food Sciences, University of Bologna, Piazza Goidanich 60, 47521 Cesena, Italy; federica.pasini5@unibo.it (F.P.); maria.caboni@unibo.it (M.F.C.); 3 Department of Nutrition and Food Science, University of Granada, Campus of Cartuja, 18071 Granada, Spain; 4 Institute of Nutrition and Food Technology ‘José Mataix’, Biomedical Research Center, University of Granada, Avda del Conocimiento sn., 18100 Armilla, Granada, Spain; 5 Dipartimento Agricoltura, Ambiente e Alimenti, Università del Molise, via De Sanctis s/n, I-86100 Campobasso, Italy; marconi@unimol.it; 6 Interdepartmental Centre for Agri-Food Industrial Research, Alma Mater Studiorum, Università di Bologna, via Quinto Bucci 336, 47521 Cesena, Italy

**Keywords:** common buckwheat, free and bound phenolic compounds, HPLC-MS, sieving, proteins, starch

## Abstract

Fractionation processes based on physical separation are a good strategy to produce enriched cereal flours. Therefore, the aim of this work is to evaluate the suitability of sieving of buckwheat flours to produce protein and phenolic (especially rutin) enriched fractions. Because of that, dehulled whole buckwheat flour (GSTQ) was sieved obtaining fractions with a particle size of 215 µm, 160 µm, 85 µm, and 45 µm (GS215, GS160, GS85, and GS45). For that purpose, the determination of protein, ash, and total starch content and free and bound phenolic compounds was carried out. The highest content of total phenolic compounds was obtained in GS215 (3118.84 mg kg^−1^ d.w.), followed by GS160 (2499.11 mg kg^−1^ d.w.), GS85 (989.46 mg kg^−1^ d.w.), GSTQ (983.15 mg kg^−1^ d.w.), and GS45 (481.31 mg kg^−1^ d.w.). Therefore, the phenolic content decreased with the particle size decrease from 215 µm to 45 µm. Besides, there were no significant differences between the total phenolic content in GS85 and GSTQ. The fraction with 215 µm reported the highest protein and mineral salt content and presented rutin amounts four times higher than GSTQ.

## 1. Introduction

Buckwheat (*Fagopyrum esculentum Moench*) is a rich source of protein, vitamins, starch, dietary fiber, and essential minerals [1,2,3]. Buckwheat also contains a high quantity of phenolic compounds, including rutin, orientin, vitexin, quercetin, isovitexin, kaempferol-3-rutinoside, isoorientin, and catechins [4]. Buckwheat contains more rutin than most of the other plants, which exhibits anti-inflammatory, antimutagenic, anticarcinogenic, antihemorrhagic, antioxidative, hypotensive, antihemorrhagic, and blood vessel protecting properties [5,6,7,8]. Phenolic compounds are presented in both free and bound forms. Whole buckwheat contains 2–5 times more phenolic compounds than oats or barley, while buckwheat bran and hulls have 2–7 times higher antioxidant activity than barley, triticale, and oats [9]. Most studies have reported that phenolic compounds are mostly bound to cell wall components in the bran and hull of most cereal grains [10]. Nevertheless, in buckwheat most phenolic compounds are found in the free form distributed throughout the entire grain (hull, seed coat, endosperm embryo axis, and cotyledons) [5,11]. The greatest concentration of these phenolic compounds is presented in the outer layers (seed coat and hull) of the grain [5]. During buckwheat flour processing, hull is removed from buckwheat seeds by impact milling and the resulting groat (or the intact achene) is roller-milled and the product is sieved to remove the fragmented hull to obtain bran flour that contains seed coat and light flour that is composed mainly of the endosperm [1,12]. One study has shown that seed coat is the part with the highest total content of phenolics from all parts of the groat [13]. Inglett et al. [11] evaluated the phenolic content in fancy (endosperm), farinetta (seed coat), supreme (whole groat), and whole buckwheat flour (whole grain), being the farinetta (seed coat) flour the most concentrated in phenolic compounds. Therefore, consumption of buckwheat flours that contents seed coat is considered to have significant nutritional or medicinal benefits [4].

The trend toward fractionation/enrichment and recombination techniques has captured the attention of the food industry in order to identify and develop green new processes respectful of the nutritional and hygienic quality of the matrix and increasing the quality of foods. In this way, separation and/or enrichment with dry fractionation technologies such as pearling/grinding, sieving, and air classification could be useful to obtain grain fractions with added value. Moreover, the products obtained with these technologies have considerable high quality in the safety point of view, compared with those obtained with other traditional methods that use the solvent extraction or chemical fractionation as enrichment process [14,15,16].

One study reported the distribution of phenolic compounds in buckwheat graded fractions, where the hull was removed from whole buckwheat grains by dehulling apparatus with disks, and the remained groats with endosperm and bran were milled to buckwheat flours and separated by weight from outer to inner parts in 16 fractions, with the fraction that contained the outermost part of the grain (bran) being the most concentrated in phenolic content [5]. It has been reported that whole grain rice flours, whole grain wheat flours, and wheat bran fours sieved with different particle size have shown different phenolic concentrations because of the different parts obtained from the buckwheat after the sieving [17,18,19]. Nevertheless, there is no study about buckwheat flour fractions from whole grain with different particle size, which would allow a gradual reduction milling system and this could be advantageous in order to obtain enriched flour fractions for the obtention of desired end-use products of high functionality [11].

For that reason, in this work the sieving of whole buckwheat flours at different particle size was carried out in order to evaluate the fractions enriched in phenolic compounds with particular attention to rutin and protein. The determination of ashes, proteins, total starch, and free and bound phenolic compounds in buckwheat flours was carried out.

## 2. Materials and Methods

### 2.1. Sample

Dehulled buckwheat grain meal (GSTQ) was obtained from buckwheat (cv. Darja) harvested in Matrice (Italy) (41°37′00″ N 14°43′00″ E), situated in a hilly location 750 m above sea level. The field presented high tenacity of the soil because of the presence of clay. Harvesting took place on September 2018. Dehulled buckwheat achenes were milled by hammer mill (model 8/B, Beccaria srl, Scarnafigi (CN), Italy); GSTQ meal was sieved to obtain four fractions with different particle size: 215 µm (GS215), 160 µm (GS160), 85 µm (GS85), and 45 µm (GS45).

### 2.2. Reagents and Chemicals

HPLC-grade acetonitrile, water, methanol, acetone, acetic acid, ethanol, hexane, ethyl acetate, diethyl ether, hydrochloric acid, sulphuric acid, ammonium sulphate, and boric acid were purchased from Merck KGaA (Darmstadt, Germany). Hydroxide sodium was from Fluka (Buchs, Switzerland). Ferulic acid, catechin, quercetin, and rutin (Sigma-Aldrich, St. Louis, MO, USA) were used for the calibration curves. Glucosidase, amyloglucosidase, peroxidase, and α-amylase were purchased from Sigma-Aldrich (St. Louis, MO, USA).

### 2.3. Protein, Ashes, and Total Starch Determination in Buckwheat Samples

#### 2.3.1. Determination of Protein

Determination of protein in buckwheat samples was carried out according to ICC method 105/2 (1995) [20]. Briefly, 1 g of sample was subjected to mineralization of organic matter with 10 mL of sulphuric acid in the presence of copper sulphate. Hence, nitrogen was changed in ammonium sulphate and treated with NaOH. The ammonia released was gathered in a solution of 4% boric acid and titrated with 0.1 N sulphuric acid.

#### 2.3.2. Determination of Ashes Content

Determination of ashes content was carried out according to ICC method 104/1 (1995) [21]. A total of 1 g of buckwheat flour was collected in a porcelain crucible in muffle furnace at 525 °C for 1 h and then cooled. After that, the sample was charred with ethanol and put in muffle at 525 °C. Ashing was completed when the cooled residue was white or nearly white. Finally, porcelain crucibles were weighed, and ashes content was calculated.

#### 2.3.3. Determination of Total Starch

The total starch in buckwheat samples was determined according to an enzymatic colorimetric method, AOAC International method 996.11 (AOAC, 2007) [22], with an assay kit from Megazyme International Ltd. (Wicklow, Ireland). Samples were ground through a 0.5-mm screen and 100.0 mg of sample was incorporated to a test tube. After that, 0.2 mL of ethanol solution (80%, *v/v*) was added into the tube and mixed to wet the sample. Then, 3 mL of thermostable α-amylase was added, and the tubes were boiled for 6 min and were shaken at intervals of 2 min. Tubes were placed in a 50 °C bath to rest for 5 min. Next, 0.1 mL of amyloglucosidase was added into each tube. Tubes were then shaken and incubated over 30 min and then filled to a volume of 10 mL with distilled water followed by centrifugation at 1800 rpm for 10 min. Then, 1.0 mL of aliquots from the supernatant was diluted in a proportion of 1/10. Next, 0.1 mL of this solution was placed into a test tube. Total of 3 mL of glucose oxidase/peroxidase reagent was added to each tube and incubated at 50 °C for 20 min. A total of 0.1 mL of water was used for blanks rather than 0.1 mL of diluted solution, and the other added reagents were all the same. Samples were read at 510 nm.

### 2.4. Extraction Methods

Extraction of free phenolic compounds from buckwheat flour fractions was carried out according to the method established by Hung and Morita [5] with certain modifications in the extraction technique and the solvent used to reconstitute the dry extract. One gram of buckwheat flour was extracted thrice in an ultrasonic bath Starsonic 90 Liarre (Bologna, Italy) equipment with frequency 34 kHz, output power (W) 190RMS, dimensions (H × W × D) 345 × 315 × 246 cm with a solution of ethanol/water (4:1 *v/v*) for 10 min. The supernatants were collected, centrifuged at 2500 rpm for 10 min, evaporated and reconstituted with 1 mL of methanol/water (1:1 *v/v*). The extracts were stored at −18 °C until analysis.

Extraction of bound phenolic compounds was carried out according to the method established by Verardo et al. (2011) [23]: Residues of free phenolic extraction were digested with 25 mL of 1M NaOH at room temperature for 18 h by shaking under nitrogen gas. The mixture was acidified (pH = 2.2–2.5) with hydrochloric acid in a cooling ice bath and extracted with 250 mL of hexane to remove the lipids. The aqueous solution was extracted five times with 50 mL of 1:1 diethyl ether/ethyl acetate (*v/v*). The organic fractions were collected and evaporated at 40 °C in a rotary evaporator. The dry extract was reconstituted in 1 mL of methanol/water (1:1 *v/v*) and stored at −18 °C until analysis.

### 2.5. Determination of Free and Bound Phenolic Compounds by HPLC- MS

A liquid chromatography apparatus HP 1100 Series (Agilent Technologies, Palo Alto, CA, USA) equipped with a degasser, a binary pump delivery system, and an automatic liquid sampler, and coupled to single quadrupole mass spectrometer detector was used. Separation of free and bound phenolic compounds from buckwheat flour fractions was carried out using a C-18 column (Poroshell 120, SB-C18, 3.0 × 100 mm, 2.7 μm from Agilent Technologies, Palo Alto, CA, USA). The gradient elution was the same as previously established by Gómez-Caravaca et al. (2014) [24] using a mobile phase A acidified water (1% acetic acid) and mobile phase B acetonitrile. MS analysis were carried out using an electrospray ionization (ESI) interface in negative ionization mode at the following conditions: drying gas flow (N_2_), 9.0 L/min; nebulizer pressure, 50 psi; gas drying temperature, 350 °C; capillary voltage, 4000 V. The fragmentor and m/z range used for HPLC-ESI/MS analyses were 80 V and *m/z* 50–1000, respectively.

Calibration curves were arranged from LOQ-500 mg/L at six concentration levels, plotting peak area vs. analyte concentration.

### 2.6. Statistical Analysis

The results of quantification reported in this work are the averages of three repetitions (*n* = 3). Tukey’s honest significant difference multiple comparison (one-way ANOVA) at the *p* < 0.05 level were evaluated by using the Statistica 7.0 software (StatSoft, Tulsa, OK, USA)

## 3. Results and Discussion

### 3.1. Yield, and Protein, Starch, and Ashes Composition in Buckwheat Samples

One of the main trends in food technologies is the use of the technological model known as fractionation/enrichment and food recombination. It consists of a preliminary extraction of constituents or enrichment of fractions (proteins, lipids, carbohydrates, fibers, flavors, dyes, etc.), which are subsequently recombined in order to obtain improved products in terms of nutritional value and dietary value. Table 1 shows the values of yield and some chemical components (protein ashes and total starch) in dehulled buckwheat flour (GSTQ) and its sieved fractions with 215 µm, 160 µm, 85 µm, and 45 µm in order to evaluate the most nutritionally adequate fraction.

As expected, the yield of GS215 and GS160 fraction is enormously lower than GS85 and GS45 fraction that correspond to the inner layers of buckwheat achene.

Protein content increased two-fold in GS215 and GS160 fractions; in contrary, it halves in GS85 and GS45 fractions. According to Schutyser et al. (2011) [25] these results confirmed that dry fractionation technologies such as sieving are a valuable tool to produce enriched protein fractions, moreover, the same authors declared that this type of technology is extremely energy efficient and is able to produce enriched fractions with retained (native) functionality compared to other green technologies such as wet fractionation.

GS 215 and GS 160 also triple the ashes content that could be related to the mineral amount; otherwise, the fractions with highest particle size showed middle content of total starch compared with GS 85 and GS45 samples.

### 3.2. Analytical Parameters of the Method Proposed

An analytical validation of the method was performed considering linearity and sensitivity. In order to quantify phenolic compounds in buckwheat fractions, five calibration curves were elaborated with the standards ferulic acid, catechin, quercetin, gallic acid, and rutin. Table 2 includes the analytical parameters of the standards used containing calibration ranges, calibration curves, determination coefficients, limit of detection (LOD), and limit of quantification (LOQ).

Calibration curves were carried out by using the peak areas of analyte standard against the concentration of the analyte for the analysis by HPLC. All calibration curves revealed good linearity among different concentrations, and the determination coefficients were higher than 0.9984 in all cases. The method used for analysis showed LOD within the range 0.0040–0.0136 mg L^−1^, the LOQ were within 0.0134–0.0452 mg L^−1^.

### 3.3. Identification of Phenolic Compounds in Buckwheat Fractions

Free and bound phenolic compounds in buckwheat flour fractions extracts were analyzed by HPLC with MS detection and were identified by rendering their mass spectra using the data reported in the literature and, when available, by co-elution with commercial standards (Table 3). A total of 32 phenolic compounds were identified in whole buckwheat flours fractions, which is in agreement with previous works [9,23,26]. Among the 32 total phenolic compounds, 25 were free phenolic compounds and 26 were bound phenolic compounds (Figure S1), identifying some of them both in free and in bound form.

### 3.4. Quantification of Phenolic Compounds in Buckwheat Fractions

A total of 25 free phenolic compounds were quantified in whole grain flour (GSTQ) and its fractions (GS215, GS160, GS85, and GS45) (Table 4). Flavonoids are the most abundant free phenolic compounds in buckwheat, which represented 73%, 66.2%, 65.6%, 75.8, and 75.8% of total phenolic content in whole grain flour and fractions (GSTQ, GS215, GS85, and GS45). The most concentrated flavonoid was epiafzelchin–epicatechin-*O*-dimethylgallate, which corresponded around 14–16% of total free phenolic compounds in whole grain flour and its fractions. The highest content of epiafzelchin–epicatechin-*O*-dimethylgallate was obtained in GS215 (225.36 mg kg^−1^ d.w.), in which the value was 58.4%, 17.3%, 63.4%, 79.5% higher than in GSTQ, GS160, GS85, and GS45. Besides that, the most concentrated phenolic acid derivative was protocatechuic-4-*O*-glucoside acid, which represented 11.1%, 15.7%, 15.4%, 9.4%, and 9.4% of the total free phenolic content, in which the highest value was obtained for GS215 followed by GS160, GSTQ, GS85, and GS45. Rutin was the second most abundant phenolic compound in whole grain flour and its sieved fractions with 45 µm and 85 µm, whereas this compound was the third most abundant in sieved fractions with 215 µm and 160 µm. Concentration of rutin in buckwheat flours decreased in the following order: GS215 > GS160 > GSTQ > GS85 > GS45 (195.47, 175.70, 87.33, 77.84, and 43.59 mg kg^−1^ d.w.).

Total free phenolic concentration decreased in the following order: GS215 > GS160 > GSTQ > GS85 > GS45. Therefore, the greatest content of free phenolic compounds was obtained in GS215 (1153.52 mg kg^−1^ d.w.), in which the value was 14.7%, 66.9%, 61.9%, and 81.5% higher than that obtained in GS160, GS85, GSTQ, and GS45.

Comparing our results of phenolic content in whole buckwheat flour (GSTQ) with previous works, Verardo et al. (2011) [23] obtained a total free phenolic content in whole buckwheat flour of 1008.91 mg kg^−1^ d.w., which was 41.43% higher than that obtained in our work. But these differences could be due to the different cultivar. Verardo et al. (2011) [23] reported that the highest free flavonoid was the rutin, whereas in our work, the most concentrated flavonoid was epiafzelchin–epicatechin-*O*-dimethylgallate followed by rutin. Kalinová et al. (2019) [13] reported the phenolic content in different parts of common buckwheat, in which the content of catechin, epicatechin, and rutin (20.87, 56.51 and 52.48 mg kg^−1^ d.w.) in groat was in the same order of magnitude as that obtained for whole buckwheat flour in our work [13]. Liu et al. (2019) [27] reported the phenolic profiles and antioxidant capacities of common buckwheat and Tartary buckwheat, in which the content of rutin in common buckwheat was 62.19 mg kg^−1^ d.w. and this value was similar to that obtained in whole buckwheat in the present study (87.33 mg kg^−1^ d.w.) [27]. Hence, our results are in accordance with the previous studies. 

Second, a total of 26 bound phenolic compounds were quantified in whole buckwheat flours (GSTQ) and its fractions (GS215, GS160, GS85, and GS45) (Table 5). Among them, flavonoids represented 63–68% of total bound phenolic content. The most concentrated flavonoid was catechin, in which the highest value was obtained in GS215 (320.22 mg kg^−1^ d.w.), followed by GS160 (241.04 mg kg^−1^ d.w.), GS85 (80.05 mg kg^−1^ d.w.), GSTQ (77.79 mg kg^−1^ d.w.), and GS45 (36.05 mg kg^−1^ d.w.). The second most abundant flavonoid was epicatechin, in which the greatest value appeared in fraction with 215 µm (202.64 mg kg^−1^), this value was 32.4, 75.3, 76.3, and 89.1% higher than that obtained in GS160, GS85, GSTQ, and GS45. Rutin was an abundant flavonoid in all fractions that represented 10–14% of total phenolic compounds, in which the highest value was obtained in GS215 (173.97 mg kg^−1^ d.w.), follow by GS160 (127.24 mg kg^−1^ d.w.), GS85 (59.09 mg kg^−1^ d.w.), GSTQ (40.09 mg kg^−1^ d.w.), and GS45 (27.09 mg kg^−1^ d.w.). The most abundant phenolic acid derivative was syringic acid, in which the greatest concentration was obtained in GS215 (100.73 mg kg^−1^ d.w.), this concentration was 21.5%, 68.9%, 69%, and 89.4% higher than that obtained in GS160, GSTQ, GS85, and GS45. There were no significant differences between the concentration of syringic acid obtained in GSTQ and GS85.

Total bound phenolic content was higher in GS215 (1565.22 mg kg^−1^ d.w.), in which the value was 25%, 69.6%, 74.9%, and 87.6% higher than that obtained in GS160, GSTQ, GS85, and GS45. Therefore, bound phenolic content decreases as the particle size falls.

By comparison of bounds phenolic compounds analyzed in whole buckwheat flours, Verardo et al. (2011) [23] reported that the total bound phenolic compounds in buckwheat was 612.33 mg kg^−1^ d.w. and this value was in the same order of magnitude as that obtained in our work. Catechin, epicatechin, and syringic acid were the most concentrated bound phenolic compounds; these results coincided with ours.

**Figure 1:**
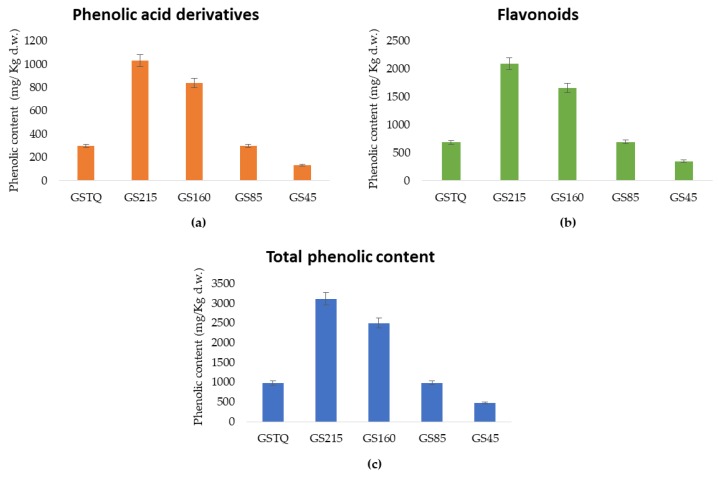
Total content of phenolic acid derivatives (**a**), total content of flavonoids (**b**), and total phenolic content (**c**) in whole grain (GSTQ) flour and its fractions (GS215, GS160, GS85, and GS45).

Figure 1 shows the sum of free and bound content of phenolic acid derivatives, flavonoids, and phenolic compounds in whole grain flour and its sieved fractions.

From total phenolic content in GSTQ and its fractions GS215, GS160, GS85, and GS45, the total phenolic acid derivatives corresponded to 27.6–33.5% of its total, in which the highest content was obtained in GS215 (1029.92 mg kg^−1^ d.w.), in which the value was 18.6%, 71%, 71.1%, and 87.1% higher than in GS160 (838.03 mg kg^−1^ d.w.), GS85 (298.61 mg kg^−1^ d.w.), GSTQ (297.96 mg kg^−1^ d.w.), and GS45 (133.07 mg kg^−1^ d.w.) (Figure 1a).

Whereas, flavonoids are the most abundant phenolic compounds in buckwheat, which represented 66.5–72.4% of total compounds in all fractions. The greatest flavonoid content in GS215 (2088.92 mg kg^−1^ d.w.) was 20.5%, 66.9%, 67.2%, 83.3% higher than that obtained in GS215, GS160, GS85, GSTQ, and GS45 (2088.92, 1661.08, 690.86, 685.19, and 348.23 mg kg^−1^ d.w.) (Figure 1b). There were no significant differences between the total content of flavonoids and phenolic acids obtained in GSTQ and GS85.

Total phenolic content was obtained in GS215 (3118.84 mg kg^−1^ d.w.), which was 19.8%, 68.5%, 68.3%, 84.5% higher than that obtained in GS160 (2499.11 mg kg^−1^ d.w.), GS85 (989.46 mg kg^−1^ d.w.), GSTQ (983.15 mg kg^−1^ d.w.), and GS45 (481.31 mg kg^−1^ d.w.). Hence, according to these results, as the particle size decreases from 215 µm there is a decrease in the phenolic content (Figure 1), this trend was similar to that obtained in previous works. Bressiani et al. (2017) [19] evaluated the total phenolic concentration in sieved whole grain wheat flours, which was higher in the fraction with the particle size of 194.9 µm (3.06 mg gallic acid/100 g flour), followed by 608.44 µm (2.23 mg gallic acid/100 g flour), 830 µm (2.11 mg gallic acid/100 g flour), and finally at 82.67 µm (1.69 mg gallic acid/100 g flour); therefore, as the particle size decreases from 194.9 µm, the phenolic content decreases. Bolea and Vizireanu (2017) [17] evaluated the phenolic content in different black rice flours that were sieved at 630, 550, 315, 180, 125, and 90 µm, the fraction with 180 µm had the highest phenolic content (483 ± 5.32 mg gallic acid/g flour), closely followed by the fraction with 315 µm (432.13 ± 7.32 mg gallic acid/g flour); whereas fractions with 125 µm and 90 µm had almost the same content (402.26 ± 8.01 and 405.32 ± 6.32 mg gallic acid/g flour, respectively). Therefore, it has been reported in the previous works that the highest phenolic content was obtained in flours sieved with a particle size of 180–194.9 µm, whose particle size was similar to our enriched fraction (215 µm), concluding that as the particle size of the fractions decreases a decrease in the concentration of phenolic compounds is obtained. This could be due to the fact that the most enriched fraction contains bran in a high proportion which possess a higher phenolic content than endosperm, and bran could be lost with the sieving at lower particle size, obtaining thereby a fine fraction which is composed mainly of endosperm that contain lower phenolic content than bran.

## 4. Conclusions

In this study, sieving was tested as a dry green technology in order to produce functional buckwheat flours. An HPLC-MS has been used for the determination of free and bound phenolic compounds in whole grain flour and its fractions sieved with 215 µm, 160 µm, 85 µm, and 45 µm of particle size. According to the results, the highest free and the bound phenolic content was obtained in buckwheat fraction with 215 µm (GS215), in which the value decreases as the particle size decreases.

Therefore, the process of milling and sieving could be used with success to increase/enrich meaningfully the content of phenolic compounds in sieved fractions from buckwheat. In fact, the concentration of rutin was 40 mg kg^−1^ d.w. in GSTQ, whereas it increased in GS215 (174 mg kg^−1^ d.w.). At the same time, the GS215 fraction reported protein and ashes amounts two times higher than the GSTQ flours.

To our knowledge, this is the first report on the use of sieving to enrich buckwheat flour with phenolic compounds (rutin among them) and protein. These preliminary results showed that this technology could be used to produce buckwheat flours, naturally enriched in proteins and phenolic compounds (rutin among others); while other fractions could be concentrated of starch. Briefly, sieved GS215 flour could be considered as naturally rich in phenolic compounds and protein buckwheat flour that could be used as an ingredient/raw material to develop functional food.

## Figures and Tables

**Table 1:** Evaluation of some chemical components (g/100 g d.w.) of dehulled buckwheat and fractions results from sieving.

	GSTQ	GS215	GS160	GS85	GS45
**Yield**	100	13.5	8.7	32.0	43.3
**Protein (N × 6.25)**	16.4 ± 0.04	35.2 ± 0.03	29.8 ± 0.04	11.3 ± 0.02	8.1 ± 0.06
**Ashes**	2.36 ± 0.003	6.05 ± 0.002	5.56 ± 0.001	1.51 ± 0.003	0.31 ± 0.002
**Total Starch**	72.6 ± 1.49	34.4 ± 1.10	43.3 ± 1.43	76.7 ± 2.00	81.2 ± 1.13

GSTQ: Dehulled buckwheat flour; GS215, GS160, GS85 and GS45: Sieved fractions with 215, 160, 85 and 45 µm, respectively.

**Table 2:** Analytical parameters of the method proposed.

Standards	Calibration Ranges (mg/L)	Calibration Curves (mg/g)	*R* ^2^	LOD (mg/L)	LOQ (mg/L)
**Ferulic acid**	LOQ-500	*y* = 119572*x* + 16157	0.9985	0.0136	0.0452
**Catechin**	LOQ-500	*y* = 170925*x* + 8609.5	0.9994	0.0095	0.0316
**Quercetin**	LOQ-500	*y* = 402162*x* + 44862	0.9996	0.0040	0.0134
**Gallic acid**	LOQ-500	*y* = 123892*x* − 4971.6	0.9984	0.0131	0.0437
**Rutin**	LOQ-500	*y* = 199694*x* − 2067.2	1	0.0081	0.0271

LOD: limit of detection, and LOQ: limit of quantification.

**Table 3:** Table of identification of free and bound phenolic compounds from whole buckwheat flour and its fractions.

Peak	Retention Time	[M–H]^−^	Molecular Formula	Compound	Free	Bound	Ion Source Fragments
1	2.07	315	C_13_H_15_O_9_	2-Hydroxy-3-*O*-β*D*-glucopyranosyl benzoic acid	+	+	
2	2.58	315	C_13_H_15_O_9_	Protocatechuic-4-*O*-glucoside acid	+	+	
3	3.22	341	C_15_H_17_O_9_	Caffeic acid hexose	N.D.	+	251
4	3.30	451	C_21_H_23_O_11_	Catechin-glucoside isomer A	+	+	289
5	4.08	341	C_15_H_17_O_9_	Caffeic acid hexose	+	+	179
6	4.17	289	C_15_H_13_O_6_	Catechin	+	+	
7	4.40	487	C_21_H_27_O_13_	Swertiamacroside isomer A	+	+	451
8	4.96	179	C_9_H_7_O_4_	Caffeic acid	+	+	
9	5.49	289	C_15_H_13_O_6_	Epicatechin	+	+	
10	6.25	561	C_30_H_25_O_11_	(Epi)Afzelchin-(Epi) catechin Isomer A	+	+	543, 425, 289
11	6.26	197	C_9_H_9_O_5_	Syringic acid	N.D.	+	
12	6.77	447	C_21_H_19_O_11_	Orientin	+	+	357
13	6.96	447	C_21_H_19_O_11_	Isoorientin	+	N.D.	
14	6.86	163	C_9_H_7_O_3_	*p*-Coumaric acid	N.D.	+	
15	7	575	C_30_H_23_O_12_	Procyanidin A	N.D.	+	289,285
16	7.46	317	C_15_H_9_O_8_	Myricetin	N.D.	+	
17	7.76	431	C_21_H_19_O_10_	Vitexin	+	+	
18	7.92	609	C_27_H_29_O_16_	Rutin	+	+	
19	7.94	441	C_22_H_17_O_10_	Epicatechin gallate	+	+	289, 169
20	7.96	833	C_45_H_37_O_16_	Epiafzelchin–epiafzelchin–epicatechin	+	N.D.	
21	8.21	451	C_21_H_23_O_11_	Catechin-glucoside isomer B	N.D.	+	289
22	8.23	487	C_21_H_27_O_13_	Swertiamacroside isomer B	+	+	451
23	8.28	463	C_21_H_19_O_12_	Hyperin	+	N.D.	
24	8.73	727	C_38_H_31_O_15_	Epiafzelchin–epicatechin-*O*-methylgallate	+	+	461, 289
25	9.31	163	C_9_H_7_O_3_	*p*-Coumaric acid	N.D.	+	
26	9.43	455	C_23_H_19_O_10_	(−)-Epicatechin-3-(3′’-*O*-methyl) gallate	+	+	289, 183
27	9.47	561	C_30_H_25_O_11_	(Epi)afzelchin-(Epi) catechin Isomer B	+	N.D.	543, 425, 289
28	9.9	757	C_39_H_33_O_16_	Procyanidin B2-dimethylgallate	+	N.D.	289
29	10.71	741	C_39_H_33_O_15_	Epiafzelchin–epicatechin-*O*-Dimethylgallate	+	N.D.	469, 319, 271
30	11.50	469	C_24_H_21_O_10_	Epicatechin-*O*-3,4-Dimethylgallate	+	+	319, 271
31	12.35	463	C_21_H_19_O_12_	Isoquercitrin	+	+	
32	12.56	301	C_15_H_10_O_7_	Quercetin	+	+	

+: detected, N.D.: not detected.

**Table 4:** Table of quantification of free phenolic compounds from whole buckwheat flour (GSTQ) and its fractions (GS215, GS160, GS85, and GS45) analyzed by HPLC-MS expressed as mg kg^−1^ d.w. flour.

Phenolic Compound	GSTQ-Free	GS215-Free	GS160-Free	GS85-Free	GS45-Free
**2-Hydroxy-3-*O*-**β ***D*-glucopyranosyl benzoic acid**	42.71 ± 1.07c	144.52 ± 1.88a	128.46 ± 2.46b	33.45 ± 1.79d	18.73 ± 1.24e
Protocatechuic-4-*O*-glucoside acid	65.56 ± 2.07c	242.95 ± 2.41a	203.55 ± 1.93b	48.50 ± 1.24d	27.16 ± 0.85e
Catechin-glucoside	23.53 ± 0.33c	45.91 ± 0.70a	40.24 ± 0.58b	22.81 ± 1.01c	12.77 ± 0.46d
Caffeic acid hexose	30.95 ± 0.74c	107.51 ± 1.55a	100.22 ± 0.92b	23.28 ± 0.36d	13.04 ± 0.19e
Catechin	27.33 ± 0.12c	72.30 ± 2.04a	64.31 ± 1.36b	21.95 ± 1.28d	12.29 ± 0.21e
Swertiamacroside	9.84 ± 0.16c	15.79 ± 1.44a	10.96 ± 1.02b	8.25 ± 0.21d	4.62 ± 0.11e
Caffeic Acid	0.01 ± 0.001c	0.06 ± 0.003a	0.024 ± 0.001b	<LOQ	<LOQ
Epicatechin	44.01 ± 1.48c	118.75 ± 3.02a	96.29 ± 2.64b	43.50 ± 1.18c	24.36 ± 0.45d
(Epi)Afzelchin-(epi) catechin isomer A	20.06 ± 1.11c	39.05 ± 0.81a	31.44 ± 0.69b	20.30 ± 1.77c	11.37 ± 1.15d
Orientin	1.58 ± 0.20c	5.64 ± 0.39a	3.12 ± 0.37b	1.00 ± 0.09d	0.56 ± 0.05e
Isorientin	0.82 ± 0.14c	3.17 ± 0.21a	1.84 ± 0.11b	0.65 ± 0.04d	0.36 ± 0.01e
Vitexin	2.02 ± 0.10c	6.00 ± 0.26a	4.11 ± 0.13b	1.49 ± 0.05d	0.83 ± 0.02e
Rutin	87.33 ± 1.11c	195.47 ± 3.62a	175.70 ± 1.87b	77.84 ± 0.94d	43.59 ± 0.51e
Epicatechin-gallate	7.22 ± 0.06c	19.44 ± 0.82a	14.81 ± 0.17b	7.65 ± 0.12c	4.28 ± 0.02d
Epiafzelchin–epiafzelchin–epicatechin	8.01 ± 0.35c	15.69 ± 0.29a	11.64 ± 0.40b	8.31 ± 0.03c	4.66 ± 0.51d
Swertiamacroside	10.17 ± 0.02c	14.59 ± 0.09a	12.76 ± 0.04b	10.76 ± 0.37c	6.02 ± 0.18d
Hyperin	1.13 ± 0.01c	3.72 ± 0.22a	1.85 ± 0.08b	0.72 ± 0.01d	0.41 ± 0.002e
Epiafzelchin-epicatechin-*O*-methyl gallate	28.73 ± 1.37c	75.39 ± 2.60a	62.88 ± 3.08b	24.31 ± 1.09d	13.61 ± 2.26e
(−)-Epicatechin-3-(3”-*O*-methyl) gallate	15.18 ± 0.10c	35.97 ± 3.58a	28.43 ± 2.19b	12.96 ± 1.43d	7.26 ± 0.88e
(Epi)afzelchin-(epi) catechin isomer B	9.95 ± 0.16c	23.25 ± 1.66a	19.29 ± 2.07b	8.62 ± 0.59d	4.83 ± 0.30e
Procyanidin B2-dimethylgallate	21.06 ± 0.08c	58.03 ± 2.01a	50.67 ± 1.59b	18.19 ± 1.27d	10.18 ± 0.64e
Epiafzelchin–epicatechin-*O*-dimethylgallate	93.83 ± 1.83c	225.36 ± 4.12a	186.37 ± 3.36b	82.38 ± 2.60d	46.14 ± 1.08e
Epicatechin-*O*-3,4-dimethylgallate	39.10 ± 0.07c	82.65 ± 1.31a	74.51 ± 2.24b	36.41 ± 1.03d	20.39 ± 0.72e
Isoquercitrin	0.46 ± 0.01d	0.72 ± 0.02a	0.63 ± 0.01b	0.58 ± 0.04c	0.33 ± 0.01e
Quercetin	0.32 ± 0.01c	1.68 ± 0.003a	1.09 ± 0.01b	0.18 ± 0.006d	0.10 ± 0.001
**Total**	**590.92 ± 13.25c**	**1553.62 ± 32.16a**	**1325.19 ± 18.14b**	**514.10 ± 10.44d**	**287.89 ± 7.91e**
**Flavonoids**	**431.68 ± 20.86c**	**1028.19 ± 19.88a**	**869.22 ± 22.09b**	**389.85 ± 15.48d**	**218.32 ± 10.72e**
**Phenolic acid derivatives**	**159.24 ± 6.48c**	**525.42 ± 11.79a**	**455.97 ± 13.93b**	**124.24 ± 8.46d**	**69.58 ± 7.11e**

Different letters (a–e) in the same line indicate significant differences (*p* < 0.05).

**Table 5:** Table of quantification of bound phenolic compounds from whole buckwheat flour (GSTQ) and its fractions (GS215, GS160, GS85, and GS45) analyzed by HPLC-MS expressed as mg kg^−1^ d.w. flour. Different letters (a–e) in the same line indicate significant differences (*p* < 0.05).

Phenolic Compound	GSTQ	GS215	GS160	GS-85	GS45
**2-hydroxy-3-*O*-**β-***D*-glucopyranosyl benzoic acid**	6.34 ± 0.08d	30.53 ± 1.22a	26.42 ± 1.43b	11.65 ± 0.04c	5.24 ± 0.02e
Protocatechuic-4-*O*-glucoside acid	4.26 ± 0.13d	18.50 ± 1.05a	15.77 ± 0.49b	8.24 ± 0.36c	3.20 ± 0.11e
Caffeic acid hexose	0.51± 0.04e	3.27 ± 0.05a	2.89 ± 0.18b	1.07 ± 0.06c	0.80 ± 0.001d
Catechin-glucoside isomer a	0.48 ± 0.01c	2.03 ± 0.04a	1.12 ± 0.01b	0.50 ± 0.02c	0.05 ± 0.0003d
Caffeic acid hexose	20.33 ± 0.12d	82.34 ± 2.10a	56.26 ± 0.86b	30.25 ± 1.15c	10.10 ± 0.46e
Catechin	77.79 ± 2.61c	320.22 ± 3.09a	241.04 ± 1.82b	80.05 ± 1.94c	36.05 ± 0.76d
Swertiamacroside	38.30 ± 3.28c	130.85 ± 1.28a	88.47 ± 1.56b	40.03 ± 2.46c	12.06 ± 0.18d
Caffeic acid	0.13 ± 0.001c	1.02 ± 0.04a	0.64 ± 0.02b	0.13 ± 0.01c	0.06 ± 0.001d
Epicatechin	47.93 ± 0.09d	202.64 ± 3.01a	136.89 ± 2.74b	50.05 ± 1.10c	22.05 ± 2.63e
(Epi)afzelchin-(epi) catechin	0.48 ± 0.03d	3.52 ± 0.11a	2.91 ± 0.004b	1.05 ± 0.01c	0.05 ± 0.002e
Syringic acid	31.28 ± 0.90c	100.73 ± 1.99a	79.03 ± 0.69b	31.26 ± 1.24c	10.72 ± 0.57e
Orientin	0.48 ± 0.02d	3.15 ± 0.09a	2.31 ± 0.003b	0.96 ± 0.01c	0.05 ± 0.003e
*p*-Coumaric acid	2.11 ± 0.10d	9.47 ± 0.11a	6.42 ± 0.30b	3.11 ± 0.14c	1.10 ± 0.02e
Procyanidin A	4.06 ± 0.08c	11.88 ± 0.32a	9.60 ± 0.24b	4.04 ± 0.07c	1.04 ± 0.04d
Myricetin	0.05 ± 0.001c	0.12 ± 0.01a	0.09 ± 0.001b	0.05 ± 0.0001c	0.01 ± 0.002d
Vitexin	3.10 ± 0.10d	14.29 ± 0.46a	11.08 ± 0.29b	5.01 ± 0.06c	2.01 ± 0.01e
Rutin	40.09 ± 2.24d	173.97 ± 2.08a	127.24 ± 1.75b	59.09 ± 0.28c	27.09 ± 1.15e
Epicatechin gallate	13.24 ± 0.69c	50.94 ± 1.30a	39.92 ± 0.84b	12.07 ± 0.45c	5.95 ± 0.28d
Catechin-glucoside isomer b	18.04 ± 0.25d	78.06 ± 0.92a	70.34 ± 1.37b	30.04 ± 0.66c	10.03 ± 0.49e
Swertiamacroside	30.04 ± 0.38d	105.31 ± 1.56a	89.39 ± 2.61b	35.07 ± 0.81c	14.05 ± 0.10e
Epiafzelchin–epicatechin-*O*-methylgallate	8.05 ± 0.11d	35.64 ± 0.86a	26.18 ± 1.27b	18.05 ± 0.78c	8.05 ± 0.04d
*p*-Coumaric acid	5.44 ± 0.44d	22.47 ± 0.19a	16.77 ± 1.06b	13.55 ± 0.07c	6.16 ± 0.86d
(−)-Epicatechin-3-(3’’-*O*-methyl) gallate	14.22 ± 0.16c	49.21 ± 0.88a	28.58 ± 1.63b	11.83 ± 0.23d	6.42 ± 0.08d
Epicatechin-*O*-3,4-dimethylgallate	1.31 ± 0.07d	5.36 ± 0.20a	3.89 ± 0.14b	2.10 ± 0.11c	0.94 ± 0.03e
Isoquercitrin	4.10 ± 0.04d	17.61 ± 0.17a	13.62 ± 1.31b	6.11 ± 0.08c	3.10 ± 0.21e
Quercitrin	20.10 ± 1.13c	92.09 ± 3.44a	77.05 ± 1.83b	20.01 ± 2.06c	7.05 ± 0.66d
**Total**	**392.23 ± 12.63d**	**1565.22 ± 14.88a**	**1173.92 ± 20.47b**	**475.37 ± 9.12c**	**193.41 ± 4.62e**
**Flavonoids**	**253.51 ± 4.80d**	**1060.73 ± 13.57a**	**791.86 ± 11.08b**	**301.01 ± 7.12c**	**129.92 ± 6.61e**
**Phenolic acids**	**138.72 ± 8.15d**	**504.49 ± 5.76a**	**382.06 ± 6.94b**	**174.36 ± 10.15c**	**63.50 ± 2.89e**

## Supplementary Materials

The following are available online at https://www.mdpi.com/2076-3921/8/12/583/s1, Figure S1: Base peak chromatogram (BPC) of bound phenolic compounds in buckwheat flour fraction GST215, obtained by HPLC-MS. See Table 3 for identification numbers.
