# Supplementary material for: Use of Sieving as a Valuable Technology to Produce Enriched Buckwheat Flours: A Preliminary Study
Source: Antioxidants (Basel). 2019 Nov 25;8(12):583. doi: 10.3390/antiox8120583 (PMC6943696; doi:10.3390/antiox8120583)

Figure S1. Base peak chromatogram (BPC) of bound phenolic compounds in buckwheat flour fraction GST215, obtained by HPLC-MS. See Table 3 for identification numbers

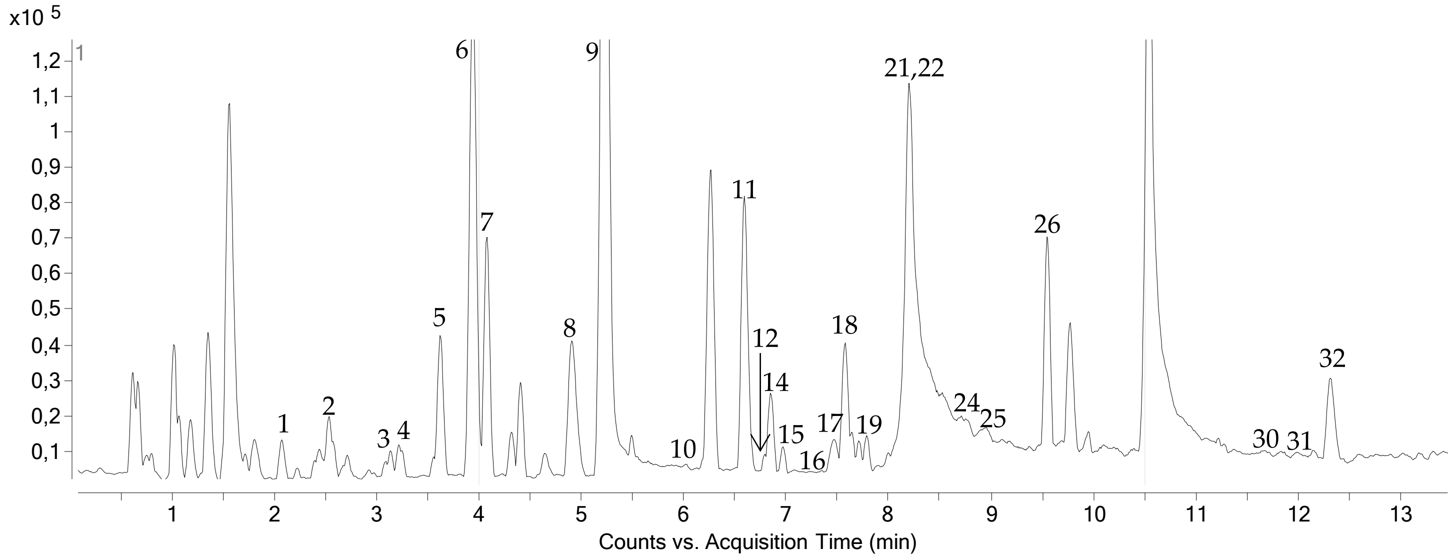

Supplement: Supplementary file 1 [file antioxidants-08-00583-s001.pdf]
